# Measles to the Rescue: A Review of Oncolytic Measles Virus

**DOI:** 10.3390/v8100294

**Published:** 2016-10-22

**Authors:** Sarah Aref, Katharine Bailey, Adele Fielding

**Affiliations:** UCL Cancer Institute, University College London, London WC1E 6DD, UK

**Keywords:** measles virus, oncolytic, virotherapy

## Abstract

Oncolytic virotherapeutic agents are likely to become serious contenders in cancer treatment. The vaccine strain of measles virus is an agent with an impressive range of oncolytic activity in pre-clinical trials with increasing evidence of safety and efficacy in early clinical trials. This paramyxovirus vaccine has a proven safety record and is amenable to careful genetic modification in the laboratory. Overexpression of the measles virus (MV) receptor CD46 in many tumour cells may direct the virus to preferentially enter transformed cells and there is increasing awareness of the importance of nectin-4 and signaling lymphocytic activation molecule (SLAM) in oncolysis. Successful attempts to retarget MV by inserting genes for tumour-specific ligands to antigens such as carcinoembryonic antigen (CEA), CD20, CD38, and by engineering the virus to express synthetic microRNA targeting sequences, and “blinding” the virus to the natural viral receptors are exciting measures to increase viral specificity and enhance the oncolytic effect. Sodium iodine symporter (NIS) can also be expressed by MV, which enables in vivo tracking of MV infection. Radiovirotherapy using MV-NIS, chemo-virotherapy to convert prodrugs to their toxic metabolites, and immune-virotherapy including incorporating antibodies against immune checkpoint inhibitors can also increase the oncolytic potential. Anti-viral host immune responses are a recognized barrier to the success of MV, and approaches such as transporting MV to the tumour sites by carrier cells, are showing promise. MV Clinical trials are producing encouraging preliminary results in ovarian cancer, myeloma and cutaneous non-Hodgkin lymphoma, and the outcome of currently open trials in glioblastoma multiforme, mesothelioma and squamous cell carcinoma are eagerly anticipated.

## 1. Introduction

Many cancers remain incurable to modern therapy despite recent pharmacological advances. Thus, there is an urgent need for the development of novel, targeted, non-toxic treatments. Oncolytic viruses are replicating viruses that preferentially infect and lyse cancer cells whilst leaving normal tissue unharmed. At least eleven viruses, including adenovirus, vaccinia virus, coxsackievirus, reovirus and measles virus (MV), are being extensively investigated and have entered clinical trials to treat a wide range of advanced cancers. Progress is highlighted by the acquisition of both the first Food and Drug Administration (FDA) licence and marketing authorization by the European Commission, for oncolytic virotherapy—talimogene laherparepvec—a herpes simplex virus that has been genetically engineered to express granulocyte macrophage colony stimulating factor (GM-CSF) to treat advanced melanoma.

The idea that replicating viruses can kill malignant cells was first suggested in the early twentieth century. In 1904, Dock [1] described two cases of chronic leukaemia that appeared to improve whilst the individual was infected by influenza; in one patient lymphadenopathy transiently reduced in size and in the other case the leucocyte count reportedly fell when infected, but both later relapsed. Reports that infection with wild type MV can have beneficial effects in cancer patients were published in the 1970s: dramatic improvements were reported in patients with acute lymphoblastic leukaemia [2], Burkitt lymphoma [3], and Hodgkin lymphoma [4]. Today, there are seven early phase clinical trials actively investigating the effect of genetically modified, vaccine strain MV to treat cancers, including multiple myeloma, head and neck cancer, and ovarian cancer. In this review, we will discuss the current understanding of the mechanisms by which MV can kill cancer cells and how this is being taken forward in the clinic as a promising therapy.

## 2. MV as an Oncolytic Virus

Safety is of paramount importance; particularly as a large proportion of cancer patients are frail and elderly. Viruses that are showing promise as oncolytic agents are either pathological strains that have been attenuated, such as vaccine strain, or alternatively they are viruses which, although they can infect and replicate in human hosts, do not cause significant disease. MV was isolated in 1954 by Enders and Peebles from a patient called Edmonston [5,6]. Following multiple passages in human kidney cells, human amnion cells and chicken embryos, a vaccine was developed; further serial passages have resulted in the strains in use today, such as Moraten and Schwarz [6,7]. These live-attenuated MV vaccine strains have been used to successfully protect millions of individuals worldwide for the past 50 years and have an excellent safety record. The vaccine can cause mild symptoms such as rash, fever and conjunctivitis in 5–15% of recipients, with reports of severe disease, or even death, being extremely rare and confined to a small number of severely immunosuppressed patients [8,9].

Any risk of the spread of a pathological strain into the general population would clearly be a cause for concern. MV has a non-segmented genome rendering it stable and at low risk of mutating, thus highly unlikely to revert to the pathogenic form. Elucidation of the MV crystal structure confirmed that the epitopes on its H envelope protein are highly conserved [10] and the MV vaccine has remained protective during the five decades of its use.

Early clinical trials of oncolytic MV are indicating that the treatment is well tolerated with only mild unwanted effects. A standard vaccine dose is not less than 1000 tissue culture infective dose 50% (TCID_50_) administered subcutaneously compared to the highest intravenous dose MV of 10^11^ TCID_50_ given intravenously within a clinical trial to treat advanced multiple myeloma. Although these were predominantly older patients who had had many courses of previous chemotherapy, and therefore a degree of frailty and immune-compromise, they tolerated the treatment well [11].

## 3. Oncolytic Measles Virus: Biological Characteristics and Cytopathic Effects

**Figure 1:**
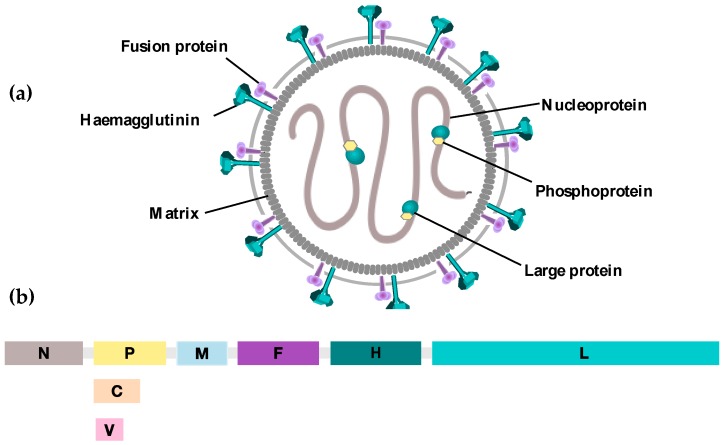
Schematic representation of (**a**) measles virus. Measles virus is an enveloped negative strand RNA virus. The RNA genome is protected by nucleoproteins (N) which are associated with a RNA dependent RNA polymerase (RdRp), known as Large Protein (L), and its cofactor Phosphoprotein (P). Together these comprise the ribonucleoprotein complex (RNP) that is surrounded by the matrix (M). The two viral glycoproteins, Haemagglutinin (H) protein and the Fusion (F) protein, project from the lipid bilayer and are involved in viral entry to the host cell; (**b**) Measles virus genome. The MV genome consists of 15,894 RNA nucleotides comprising six transcription units each separated by trinuclear intergenic sequences. A transcriptional gradient is generated whereby mRNAs are generated with decreasing abundance from the 3′ N to the 5′ L position. The proteins V and C are non-structural proteins that are generated from an alternative RNA transcript of the P gene.

MV is an enveloped single-stranded negative-sense RNA virus belonging to the *Morbillivirus* genus and the family of *Paramyxoviruses*. The 16 kilobase long genome comprises six genes that encode eight viral proteins. The viral genome is encapsidated by nucleoprotein (N), phosphoprotein (P) and large protein (L) forming the ribonucleoprotein complex (RNP), which is surrounded by matrix (M) protein. Two of the proteins are non-structural proteins V and C, expressed from an alternative RNA transcript of the P gene. Their role is primarily implicated in the prevention of type 1 interferon (IFN)-induced immune responses. MV envelope glycoproteins hemagglutinin (H) and fusion (F) proteins mediate virus attachment and fusion, respectively [12,13] (Figure 1).

MV infection is initiated by the interaction of its H protein with cell surface receptors. This interaction induces a conformational change that activates the viral F protein, which mediates membrane fusion at neutral pH to permit the entry of RNP into the target cell cytoplasm. Expression of the F and H proteins on the surface of infected cells subsequently leads to their fusion with neighbouring non-infected cells to promote efficient viral spread. This cell-to-cell fusion gives rise to giant multinucleated cells, called “syncytia”—the cytopathic hallmark of MV infection [14,15]. Syncytia are metabolically active and their formation is correlated with improved virus-mediated cytotoxicity both in vitro and in vivo [16,17].

## 4. MV Receptors

Three main receptors, CD150, CD46 and nectin-4, are utilized by MV for target cell entry. CD150 or signalling lymphocyte activation molecule (SLAM) is the main receptor for wildtype MV strains [18]. The receptor is predominantly expressed on activated B and T lymphocytes, immature thymocytes, monocytes and dendritic cells (DCs) [19,20,21,22]. On the contrary, attenuated vaccine strains of the MV-Edm lineage, which are selectively oncolytic, have adapted to use CD46 receptor through a single amino acid substitution at position 481 in MV-H protein (asparagine to tyrosine) [23]. The type I transmembrane glycoprotein CD46 acts as a negative regulator of complement-mediated lysis and is ubiquitously expressed in all nucleated cells [24,25].

A more recently identified epithelial receptor, nectin-4, is exploited by both wildtype and vaccine strains to facilitate cellular entry [26,27]. Nectin-4 is an adherens junction protein, originally described as poliovirus-receptor-like-4 (PVRL-4), and plays an important role in MV pathogenesis by ‘shedding’ the virus in epithelial airways through the course of infection [28].

## 5. Mechanisms of Specificity

### 5.1. Relationship of Receptor Expression to Oncolytic Activity

In relation to MV-mediated oncolysis, the frequent overexpression of CD46 on many tumour cell types to escape autologous complement-dependent cytotoxicity accounts for some of the selectivity of attenuated MV replication in transformed cells [29]. Furthermore, recent studies have demonstrated that MV-Edm strains retain their ability to use SLAM as a receptor. In fact, MV-mediated oncolysis of primary mantle cell lymphoma cells and xenografts has been shown to be SLAM-dependent and not correlated to CD46 expression levels [30].

Moreover, whilst MV receptor nectin-4 is expressed at low to moderate levels in respiratory epithelial cells, a number of studies have reported its abundant expression in lung, colon, ovarian and breast adenocarcinomas, making it a potential tumour marker [31,32,33]. The increasing anti-tumour activity of MV-Edm in human adenocarcinoma cells with up-regulated levels of nectin-4 suggested that its expression is correlated with MV-mediated oncolysis [34,35].

### 5.2. Defects in Interferon Response

Apart from MV tropism to specific cellular receptors, other potential mechanisms also contribute to the tumour selectivity of MV vaccine strains. Defects in the IFN anti-viral response pathway are common in tumour cells, making them more permissive to viral infections compared to their normal counterparts [36]. In normal MV-resistant cells, sensing of viral RNA by pattern recognition receptors (PRRs), RIG-I and MDA-5 activates a cascade of innate immune pathways that trigger the production of type 1 interferon (IFNα/β). IFN then binds to its cognate receptor to activate the Janus kinase/signal transducers and activators of transcription (JAK/STAT) pathway, which subsequently results in the induction of IFN-stimulated genes (ISGs) involved in anti-viral defence and apoptosis signals. This pathway is frequently deregulated in tumour cells to facilitate their escape from the host immune system [37]. A study investigating the role of the type 1 interferon pathway in MV-permissiveness demonstrated that pre-treatment of MV-susceptible sarcoma cell lines with exogenous IFN-β renders them more resistant to MV-mediated oncolysis; suggesting that their differential susceptibility to MV infection may be attributed to their different innate immune responses [38].

## 6. Role of the Immune System in MV-Mediated Oncolysis

Another attraction of virotherapy is the ability to harness the immune system to recognize and target infected cancer cells for destruction—with a possible bystander effect. The immune system may play a role in augmenting therapy with oncolytic MV. However, this has proved challenging to study due to the lack of in vivo murine models that are susceptible to MV infection and at the same time have intact immunity. A pivotal role for innate immunity in MV-mediated anti-tumour effects has been demonstrated in studies where treatment of Burkitt’s lymphoma xenograft models with MV expressing murine granulocyte macrophage colony-stimulating factor (GM-CSF) was associated with neutrophil infiltration and tumour regression, significantly enhancing the therapeutic potential of the virus compared to the parental one [39]. Another study evaluating the role of neutrophils showed that neutrophil depletion abrogated the therapeutic effect of MV expressing human granulocyte colony-stimulating factor (GCSF) in a Burkitt’s lymphoma in vivo mouse model [40]. Other key players of the innate immune system, dendritic cells (DCs), have also been implicated in contributing to oncolytic MV therapy. Plasmacytoid DCs (pDCs) exposed to MV-infected tumour cells showed in vitro maturation, enhanced production of IFNα and cross-presenting of tumour antigens to CD8+ T-cells leading to immunogenic cell death [41]. Furthermore, MV infection of melanoma cells has been shown to activate DCs by upregulating costimulatory surface activation markers CD80 and CD86, thus stimulating an adaptive anti-tumour immune response [42].

## 7. Engineering Oncolytic Measles Virus

### 7.1. Targeting MV Entry

Despite its natural tropism to cancer cells, numerous strategies have been developed to re-direct MV specificity to cancer cells, thereby minimizing off-target side effects and addressing further safety concerns of administering higher viral doses. Cellular entry of MV is majorly determined by the interaction between MV-H protein and MV cell surface receptors. Modifications of MV tropism have been achieved by the insertion of tumour-specific ligands at the carboxyl-terminal extensions of the H protein. Single chain antibodies against tumour associate antigens CEA, CD20 and CD38 have all been displayed on recombinant MV to facilitate targeted entry to epithelial carcinoma, non-Hodgkin’s lymphoma and myeloma cells, respectively [43,44,45]. Furthermore, engineering measles virus that expresses integrin-binding peptides, cyclic-arginine-glycine-aspartate (RGD) [46] or echistatin [47], has successfully retargeted the virus to endothelial cells of tumour neovessels. Attenuated measles virus, engineered to express high affinity single chain T-cell receptors (scTCR) can be retargeted to specific major histocompatibility complex (MHC) ligands [48]. In the studies described above, the native binding of attenuated MV to CD46, SLAM or nectin-4 was retained to allow viral entry to tumour cells expressing those receptors. Amino acid residues necessary for the interaction of MV-H protein with its native receptors have been well characterized thus allowing the generation of selectively receptor-blind MV that gains cellular entry via alternative non-native receptors. Ablation of CD46 and SLAM binding has been achieved by single amino acid mutations, Y481A and R533A, respectively. Moreover, MV can become nectin-4-blind via a single amino acid substitution (Y543A) in the wildtype MV-H glycoprotein [28]. Those truly retargeted viruses can no longer bind native MV receptors and are stably maintained through multiple passages [49]. The mutant viruses are rescued and propagated in a system that uses Vero-a-His cells expressing a membrane-bound single chain antibody. Upon recognition of a six histidine peptide (H6) in the C-terminal end of the receptor-blind MV-H protein, the scFv is incorporated in that site. This “pseudoreceptor” system was used to rescue and propagate receptor-blind MV displaying scFvs against EGFR, mutant EGFR, and CD38 between their H6 peptides and the mutated H-protein [50].

Additional methods to achieve MV oncotropism involve modification of its fusion properties. MV fusion protein is initially synthesized as an inactive precursor F0. Activation of F0 requires proteolytic cleavage into F1 and F2 subunits mediated by the ubiquitously expressed cellular protease furin [51]. Furin recognizes MV-F through a cleavage site composed of five basic amino acids (Arg–Arg–His–Lys–Arg) [52]. Indeed, it has been demonstrated that MV-F proteins with mutated cleavage sites that depend on activation by tumour-specific-proteases rather than by furin, can restrict virus activation to cancer cells [53,54,55]. Cancer cells are characterized by their secretion of matrix metalloproteinases (MMPs)-enzymes that play an essential role in cancer cell invasion and metastasis by degrading extracellular matrix proteins. The substitution of the furin-cleavable site of the viral F glycoprotein with MMP-cleavable sequences conferred the virus selective fusogenicity with MMP-expressing cancer cells [54].

Alternative approaches to retarget MV at a post-entry level have been developed by expressing synthetic microRNA (miRNA) targeting sequences in the 3′UTR of the MV fusion protein [36,56]. Endogenous cellular miRNAs are frequently downregulated in tumour cells compared to normal cells. In this way, viral replication is specifically repressed in normal cells, thereby improving tumour specificity without compromising oncolytic efficiency. In this manner, a miRNA-sensitive measles virus was engineered to target miRNA-7, which is reportedly downregulated in gliomas. While the potency of the oncolytic virus was retained in glioblastoma multiforme xenografts, viral replication was repressed in normal brain tissue, where miRNA-7 is ubiquitously expressed [35]. Moreover, to achieve “detargeting” of multiple vital organs during the systemic administration of oncolytic MV, a triple-miRNA-sensitive virus was developed targeting miRNA-7, miRNA-122 and miRNA148a, commonly expressed in brain tissues, liver and gastrointestinal organs, respectively [57].

### 7.2. Monitoring and Tracking Viral Replication

Although, oncolytic MV is able to preferentially infect and replicate in tumour cells, there remains an advantage to engineering “trackable” viruses. Various non-invasive measures have been developed to monitor in vivo viral replication and kinetics. In the clinical settings, this has provided critical information for the optimisation of therapeutic protocols by determining suitable viral doses and time intervals between repeated treatment cycles [58]. Recombinant MV-Edm strains encoding green fluorescent protein (GFP) and the soluble extracellular domain of human carcinoembryonic antigen (CEA) sequences at the 3′ end of the MV genome, upstream of MV-N protein, have been generated to facilitate real-time viral gene expression profiles. The insertion of reporter gene sequences at this position of the viral genome ensures high levels of gene transcription. MV-GFP is used to visualize MV-induced cell fusion studies in vitro [59] and in vivo [60]. CEA is a well-characterized soluble peptide routinely used as a tumour marker for certain human cancers [61]. Infection of cancer cells with MV-CEA releases CEA into the blood stream, allowing the subsequent detection of CEA levels in the serum of treated patients [58].

Another “trackable” MV is one that expresses thyroidal sodium iodide symporter (NIS) downstream of the MV-H protein (MV-NIS) [62]. NIS is a membrane ion channel that mediates iodine uptake and concentration in the thyroid gland [63,64]. MV-NIS infection of cancer cells followed by administration of iodide isotope tracers such as I123, I124 and Tc99M results in increased intracellular concentration of the isotope, thereby allowing the non-invasive in vivo detection of viral localisation and spread over time by gamma camera imaging, PET or SPECT/CT [65]. One disadvantage with the use of reporter genes is the inability to discriminate between infection in cancer and normal cells. A recombinant MV-Edm expressing human lambda light immunoglobulin chain (IgG-λ) as a reporter gene upstream of MV-N protein was generated to infect multiple myeloma (MM) cells [66]. Over-production of monoclonal kappa light immunoglobulin (IgG-κ) is a major hallmark of MM. Infection of MM cells with this virus strain (MV-lambda results in the secretion of unique chimeric immunoglobulin consisting of one kappa and one lambda light chain. This converted marker molecule is not naturally present in vivo and can be quantified using immunoassay methods. On the contrary, MV-lambda-infected normal non-myeloma cells secreted only free lambda light chain and not the chimeric immunoglobulin. This approach of “marker conversion” thus allows the production of cancer-specific viral infection markers [66].

### 7.3. Enhancing Oncolytic MV Activity

#### 7.3.1. “Radiovirotherapy”

Besides tracking MV replication by MV-NIS, expression of NIS increases virotherapeutic efficacy by facilitating the intracellular entry of beta-emitting radioisotopes such as I131 and Re188, specifically inducing radiation damage within the tumour microenvironment [62,67,68]. Radiovirotherapy of in vivo multiple myeloma models with relatively small doses of MV-NIS followed by iodine-131 subsequently resulted in the eradication of infected tumour cells, which are otherwise resistant to MV-mediated lysis [62].

#### 7.3.2. “Chemovirotherapy”

In an approach to combine virotherapy with chemotherapy, oncolytic MV strains have been generated to express prodrug convertases or “suicide” genes that catalyse the conversion of chemotherapeutic prodrugs into highly toxic metabolites. These metabolites are then incorporated into the DNA of replicating cells, inducing apoptosis in infected cells. This strategy of prodrug convertase transgene-mediated enhancement of oncolytic activity is known as “chemovirotherapy”. *Escherichia coli* purine nucleoside phosphorylase (PNP) is a prodrug convertase that converts chemotherapeutic prodrugs fludarabine and 6-methylpurine-2′-deoxyriboside (MeP-dR) into highly toxic 2-fluoroadenine and 6-methylpurine (MeP), respectively. The expression of PNP by a CD20-retargeted MV has demonstrated enhanced therapeutic efficacy in murine models of Burkitt’s lymphoma after fludarabine administration [69]. Moreover, intravenous infection with MV-antiCEA-PNP following MeP-dR administration showed enhanced anti-tumour effects with significant survival rates in syngeneic colon adenocarcinoma xenografts [70]. Another prodrug convertase expressed by oncolytic MV is super-cytosine deaminase (SCD), encoding a fusion protein comprising of yeast cytosine deaminase and yeast uracil phosphoribosyltransferase. SCD converts the prodrug 5-fluorocytosine (5-FC) to 5-fluorouracil (5-FU) and finally to 5-fluorouridine-monophosphate. Co-administration of MV-SCD and 5-FC has proved to enhance anti-tumour efficacy in cholangiocarcinoma, hepatocellular carcinoma, melanoma and ovarian cancer xenografts models [71,72,73,74].

#### 7.3.3. “Immunovirotherapy”

Strategies to enhance the oncolytic efficacy of measles virus have been developed by insertion of immuno-modulatory transgenes that stimulate the native anti-tumour immune response. In immune-deficient murine models of human B-cell lymphoma and colon adenocarcinoma, treatment with a GM-CSF-expressing MV significantly enhanced tumour regression or delayed tumour regression, which was correlated with an influx of host neutrophils and tumour-infiltrating CD3+ T-lymphocytes [39,75]. Oncolytic MV expressing murine interferon beta (mIFNβ) gene triggered innate immune cell infiltration and slowed tumour growth and angiogenesis in human mesotheliomas xenografts [76]. The expression of *Helicobacter pylori* neutrophil-activating protein (NAP), by attenuated MV has also been shown to induce a potent anti-tumour immune response in lung and intrapleural metastatic breast cancer xenograft models via stimulating the release of proinflammatory cytokines [77]. MV has also been engineered to code for other immune-stimulating transgenes including interleukin (IL-13) [78] and heat shock protein inhibitors [79].

A more recent approach to generate immune-armed oncolytic MV was achieved by incorporating antibodies against immune checkpoint inhibitors. T cell proliferation is precisely regulated by maintaining a balance between lymphocyte activation and suppression via co-stimulatory and co-inhibitory signals, respectively. This is critical to ensure effective immune responses while preventing uncontrolled T cell proliferation and autoimmune damage to non-target tissues. Cytotoxic T-lymphocyte antigen-4 (CTLA-4) and programmed cell death-1 (PD-1) are inhibitory receptors that limit T cell activation. Tumour cells have been demonstrated to exploit this mechanism of T cell ablation to evade the immune system. Thus, FDA-approved antibodies blocking CTLA-4 and PD-1 and its ligand PD-L1 show promising anti-tumour effects in a wide range of tumour types by priming T cells against tumour antigens [80,81]. The combination of oncolytic MV with antibodies targeting CTLA-4 (MV-αCTLA-4) and PD-L1 (MV-αPD-L1) demonstrated enhanced efficacy in immuno-deficient MV-susceptible melanoma mouse models. Treatment with both viruses resulted in delayed tumour progression. Prolonged overall survivals were observed in mice treated with MV-αPD-L1. This was associated with a significant increase in cytotoxic T cells and a decline in regulatory T cells infiltration within tumours [82]. The genetic modifications of MV are summarised in Table 1.

## 8. Overcoming Anti-Viral Host Immune Responses

Following successful worldwide vaccination programs, the majority of the population is immune to measles. Thus, there is concern that pre-existing neutralizing antibodies to measles will diminish or prevent oncolysis. This is particularly problematic for intravenous therapy where even relatively low titres of antiviral antibodies can negate the anti-tumour effect [110]. There are different strategies to overcome this problem.

### 8.1. Concomitant Immunosuppressive Therapy

Cyclophosphamide is a commonly used alkylating agent used both as an anticancer chemotherapy and as an immunosuppressant—lymphocytes, including B- and T-cells are exquisitely sensitive to cyclophosphamide [111]. In CD46 transgenic immunized mice cyclophosphamide can effectively reduce the anamnestic response [112]. This approach is currently being tested in myeloma clinical trials [113].

### 8.2. Shielding from Immune Attack

Another approach to evade the host’s neutralising anti-measles antibodies is to hide the virus whilst it is transported to the site of cancer cells—a cell-based delivery system—this has been effective in pre-clinical models using mesenchymal stromal cells (MSCs). MSCs are plastic-adherent fibroblast-like cells that have a high self-renewal capacity, multilineage potential and also have immunomodulatory properties [114]. The strategy involves in vitro infection of MSCs with MV, and effective targeting to the tumour site. This approach is effective in pre-clinical models of acute lymphoblastic leukaemia [115], hepatocellular carcinoma [116] and ovarian carcinoma [117]. A phase 1 clinical trial is currently recruiting patients with ovarian cancer to investigate the use of MSCs of adipose origin to target MV to the transformed cells.

An alternative strategy to “hide” from the immune system is to protect the virus in a coat of nanoparticles. Core-shell type iron oxide magnetic nanoparticles have been shown to form stable complexes with viruses and have been assembled with adenovirus and vesicular stomatitis virus. To the authors’ knowledge, this remains restricted to in vitro studies. The particles also offer the means to monitor the virus in vivo with magnetic resonance imaging (MRI) imaging [118]. Creating a chimeric virus by exchanging its envelope glycoproteins with the closely related canine distemper virus—a morbillivirus, to which humans are not already immune—is yet another approach to evade host immunity [119].

## 9. Clinical Trials

The first clinical trial of oncolytic MV was a small open-label Swiss study enrolling five patients with cutaneous T-cell lymphoma stage IIb or higher, resistant to, or relapsing following conventional therapy. Edmonston-Zagreb strain (MV-EZ) MV was injected into the tumour following treatment with subcutaneous IFN-alpha. Each treatment cycle consisted of two separate MV doses. This was a dose escalation study using relatively low doses of MV from 100 to 1000 TCID_50_. At day 28, five of a total six lesions had regressed and one disappeared completely. In two patients, distant lesions also improved despite treatment being localized. One patient had progressive disease. Adverse effects were limited to grade 1, indicating safety of the treatment. This was encouraging, particularly given that the effects of the virus could spread from the injection site—as seen by the improvement of the distant tumour [84].

Ovarian cancer accounts for approximately 3% of cancers in women in the USA, but causes a greater number of deaths than other malignancies of the female genital tract (CDC). Ovarian cancer not only expresses the MV receptor CD46 at high levels, but also the more recently discovered nectin-4 [31]. Galanis et al. (2010) [92] recruited 21 patients with recurrent, or progressive ovarian cancer or primary peritoneal cancer, all of whom had been previously treated with platinum based chemotherapy plus Taxol. Although this is no longer the case in MV clinical trials, patients had to have proven immunity to MV prior to enrolment. In this trial, MV immunity was determined by anti-measles IgG levels ≥ 20 ELISA units/mL by enzyme immunoassay. In order to monitor infectivity, the MV Edmonston vaccine strain was engineered to express human carcinoembryonic antigen (CEA)—an oncofetal antigen that is produced in trace amounts by normal adult cells and is expressed in increased amounts in adenocarcinomas. That CEA is not normally expressed in ovarian cancer and there is a widely available routine laboratory immunoassay makes it an attractive modification. MV-CEA was injected into the intraperitoneal cavity in four weekly schedules to a maximum of six cycles with doses ranging from TCID_50_ 10^3^ to 10^9^, but no dose limiting toxicity was observed. Median overall survival was 12.15 months—this compared favourably to the expected survival at the time of publication. Increased levels of CEA were measured in the serum in only three patients, all of whom had received the highest 10^9^ TCID_50_ dose. A further clinical trial in ovarian cancer abandoned the MV-CEA in favour of MV engineered with the sodium iodine symporter (NIS) as means to monitor tissue that has been infected with the virus. Positioning the NIS transgene downstream of the haemagglutinin (H) gene, as opposed to upstream of the N gene in the MV-CEA construct, they argued provided an advantage in viral proliferation, which also facilitates viral manufacture [120]. In this clinical trial only the higher doses of MV TCID_50_ 10^8^ and 10^9^ were used for the 16 recruited patients. ^123^I SPECT/CT imaging was performed to monitor the NIS transgene, however uptake was observed in only three of the 13 patients treated at the highest MV dose. A favourable median overall survival of 26.6 months was reported for this heavily pre-treated group of patients.

There are a number of currently open and recruiting MV clinical trials, including for patients with glioblastoma multiforme (NCT00390299), mesothelioma (NCT01503177), squamous cell carcinoma of the head and neck (NCT01846091), peripheral nerve sheath tumour (NCT02700230) multiple myeloma (NCT02192775, NCT00450814), ovarian, fallopian and peritoneal cancer (NCT00408590, NCT02364713). An interim report details the remarkable improvement of two heavily pre-treated patients with myeloma. This was the first report of the use of intravenous MV and supports the safety of systemic administration at a dose of 10^11^ TCID_50_. The dramatic improvement seen in these patients can be explained, at least in part, by their low titres of anti-measles antibodies [11]. A further study in ovarian cancer (NCT02068794) also uses adipose-derived mesenchymal stem cells to “transport” MV to the target cells.

## 10. Conclusions

MV has been extensively investigated as an oncolytic therapy in the pre-clinical setting and is showing promise in on-going and completed clinical trials. The excellent safety record of the vaccine strain coupled with the ability to engineer the viral genome to effectively target the virus to malignant cells and monitor its progress makes it an exciting candidate. The lymphotropic nature of oncolytic MV makes it an attractive option, particularly in lymphoid malignancies.

On-going efforts to address the issue of pre-existing anti-MV antibodies in immunized patients have shown encouraging results in the pre-clinical settings and results from clinical trials are pending. A combination of MV with other immune therapies will undoubtedly be of interest and advances in the understanding of the interplay between oncolytic MV and the tumour microenvironment may further improve its therapeutic outcome. Finally, although a number of mechanisms have been determined for MV-mediated oncolysis, the exact mechanisms remain elusive. Developments in this area will be vital for future directions in the use of MV as an oncolytic agent.

## Figures and Tables

**Table 1:** Summary of genetically engineered MV.

	Virus Strain	Genetic Modification	Tumour Type
**Unmodified attenuated vaccine strain**	MV-Edm	Unmodified	Leukemias/lymphomas [69,83,84], multiple myeloma [85], ovarian cancers [86], breast cancer, melanoma, renal cell carcinoma, fibrosarcoma
**Retargeted virus**	MV-CD20	Retargeted to CD20	Leukemias, fibrosarcoma [43]
	MV-CD38	Retargeted to CD38	MM, Erythroleukemia, Burkitt’s lymphoma, ovarian cancer, GBM [50]
	MV-HER2/neu	Retargeted to her2/neu	Ovarian cancer, medulloblastoma [87]
	MV0EGFRvIII	Retargeted to EGFR	Erythroleukemia, Burkitt’s lymphoma, ovarian cancer, GBM [66]
	MV-PSMA	Retargeted to PSMA	Prostate cancer [88]
	MV-CD133	Retargeted to CD133	Hepatocellular carcinoma, colon, glioma [89]
	MV-aVb3-integrin targeted	Retargeted to aVb3-integrin	Multiple myeloma [47]
	MV-MMP	MMP-activated virus	Hepatocellular carcinoma, fibrosarcoma, cholangio carcinoma [54,55]
	MV-miRNA-sensitive	Retargeted to miRNA sequences	GBM [56]
	MV-DARPins	Retargeted to EGFR, Her2/neu and EpCAM	Adenocarcinoma, breast ductal carcinoma, colon adenocarcinoma, fibrosarcoma, ovarian carcinoma, glioblastoma [90]
**Reporter genes**	MV-CEA	CEA reporter gene	Ovarian cancer [91,92], GBM [93,94], breast cancer [95], hepatocellular carcinoma [96], prostate cancer [97], rhabdomyosarcoma [79]
	MV-NIS	NIS reporter gene	Ovarian cancer [91], MM [98], GBM [99], hepatocellular carcinoma [96], pancreatic cancer [100], prostate [97], head oral squamous cell carcinoma, anaplastic thyroid carcinoma, hypopharyngeal carcinoma [101], colorectal cancer [102], endometrial cancer [103], T cell lymphoma [104]
	MV-LacZ	Beta-galactosidase reporter gene	Lymphoma [105]
	MV-lambda	Human light immunoglobulin chain reporter gene	MM [106]
**Suicide genes**	MV-PNP	Prodrug convertase	Leukemia/lymphoma [107], colorectal cancer [70], pancreatic cancer [108]
	MV-SCD/FCU1	Prodrug convertase	Ovarian cancer [71], melanoma [72], cholangiocarcinoma [73], hepatocellular carcinoma [74], colorectal carcinoma [109]
**Immuno-stimulating genes**	MV-GMCSF	GM-CSF gene	ALL cells, Burkitt’s lymphoma [39,75]
	MV-NAP	Neutrophil activating protein gene	Breast cancer [77]
	MV-alphaCTLA4	Retargeted to CTLA-4 antibody	Malignant melanoma [82]
	MV-alphaPDL-1	Retargeted to PDL-1 antibody	Malignant melanoma [82]
	MV-Hblind-IL13	Retargeted to IL13	GBM [78]
	MV-IFN	IFN immunomodulatory gene	Mesothelioma [76]

GBM: glioblastoma multiforme; MM: multiple myeloma; CD: cluster of differentiation; HER2/neu: human epidermal growth factor receptor 2; EGFR: epidermal growth factor receptor; PSMA: prostate-specific membrane antigen; MMP: matrix metalloproteinase; miRNA: microRNA sequences; DARPin: designed ankyrin repeat proteins; CEA: carcinoembryonic antigen; NIS: sodium iodide symporter; PNP: purine nucleoside phosphorylase; SCD: super cytosine deaminase; GM-SCF: granulocyte macrophage colony stimulating factor; NAP: neutrophil-activating protein; CTLA-4: cytotoxic T lymphocyte antigen 4; PDL-1: programmed death-1 ligand 1; IL: interleukin; IFN: interferon beta.
